# Interface Quality Control of Self-Assembled Monolayer for Highly Sensitive Protein Detection Based on EGOFETs

**DOI:** 10.3390/s26082290

**Published:** 2026-04-08

**Authors:** Xinyu Dong, Xingyu Jiang, Jiaqi Su, Zhongyou Lu, Cheng Shi, Dianjue Liu, Lizhen Huang, Lifeng Chi

**Affiliations:** 1 State Key Laboratory of Bioinspired Interfacial Materials Science, Institute of Functional Nano & Soft Materials (FUNSOM), Soochow University, Suzhou 215123, China; 20234214162@stu.suda.edu.cn (X.D.); xyjiang@suda.edu.cn (X.J.); zyluzy@stu.suda.edu.cn (Z.L.); djliu2819@stu.suda.edu.cn (D.L.); 2 School of Integrated Circuits, Southeast University, Nanjing 210096, China; 220236459@seu.edu.cn; 3 Macao Institute of Materials Science and Engineering (MIMSE), MUST-SUDA Joint Research Center for Advanced Functional Materials, Macau University of Science and Technology, Taipa, Macao 999078, China; shicheng1708404032@163.com

**Keywords:** EGOFETs, immunoglobulin G, self-assembled monolayer, interface engineering

## Abstract

Biosensors based on electrolyte-gated organic field-effect transistors (EGOFETs) have attracted considerable attention due to their advantages, including low cost, inherent signal amplification, and low-voltage operation. A critical step influencing sensing performance is the integration of specific receptors onto the device surface. Among various strategies, the covalent immobilization of biorecognition elements onto gold surfaces via thiol chemistry is one of the most widely used approaches. In this study, we report the optimization of a mixed self-assembled monolayer (SAM) composed of 11-mercaptoundecanoic acid (11-MUA) and 3-mercaptopropionic acid (3-MPA) for label-free detection of human IgG using EGOFETs. The quality of the SAM was systematically modulated by varying the total concentration from 10 to 400 mM and characterized using X-ray Photoelectron Spectroscopy (XPS), Electrochemical Impedance Spectroscopy (EIS), Cyclic Voltammetry (CV), and Atomic Force Microscopy (AFM). The results revealed that a concentration of 50 mM yielded a densely packed and well-ordered monolayer. After covalent immobilization of anti-IgG antibodies via 1-ethyl-3-(3-dimethylaminopropyl)carbodiimide hydrochloride/N-hydroxysuccinimide (EDC/NHS) chemistry and subsequent blocking with ethanolamine and bovine serum albumin (BSA), the functionalized gate electrodes were integrated into poly(3-hexylthiophene) (P3HT)-based EGOFETs. Electrical measurements demonstrated that EGOFET biosensors functionalized with the 50 mM SAM achieved optimal sensing performance. The devices exhibited a highly linear response (R^2^ = 0.998) over a wide concentration range from 1 fM to 10 nM, with a LOD of 2.82 fM, and showed excellent selectivity against non-target immunoglobulins A and M (IgA and IgM). This SAM concentration optimization strategy provides a versatile approach for engineering high-performance EGOFET biosensors, with potential applicability to a broad range of disease biomarkers.

## 1. Introduction

Immunoglobulin G, IgG, the most abundant serum antibody [1,2,3], plays a critical role in disease diagnosis and immunological evaluation. However, conventional detection methods, such as enzyme-linked immunosorbent assays [4,5,6] and colloidal gold lateral flow immunoassays [7,8], often face a trade-off between accuracy and convenience, while traditional electrochemical biosensors are limited by insufficient signal amplification [8]. Electrolyte-gated organic field-effect transistors (EGOFETs) present a promising alternative, combining low cost, inherent signal amplification, and low-voltage operation to enable the development of highly sensitive, rapid, and user-friendly IgG detection platforms [9,10,11,12,13].

The core of EGOFET-based biosensors lies in the effective integration of biological components (such as proteins, DNA, or RNA) with device structures [14,15,16], including the gate, electrolyte, and channel. Among various strategies, gate-surface functionalization has become the most prevalent approach. Au gates are widely used as the sensing interface in existing sensing systems [17,18,19]. Under this framework, the covalent immobilization of biorecognition elements onto gold surfaces via thiol chemistry [20,21,22] has gained the widest application. In particular, a mixed self-assembled monolayer (SAM) has been extensively demonstrated to enable dense and functionally oriented immobilization of bioreceptors on gold [17,23,24,25]. This strategy has shown outstanding performance in detecting targets across different scales. For small-molecule detection, Casalini et al. [26] used a mixed SAM composed of cysteamine and 4-formylphenylboronic acid to selectively capture dopamine, achieving a limit of detection (LOD) of 0.1 nM. In the field of macromolecular detection, such as proteins, the Toris group [27] reported an EGOFET-based single-molecule immunosensor modified with a hydrogen-bond networked SAM. By immobilizing anti-IgG on this mixed SAM containing amide-based hydrogen-bonding motifs, label-free detection of single IgG molecules in diluted saliva was realized. Furthermore, Berto et al. [28] developed the EGOFET immunosensor for detecting anti-drug antibodies (ADAs), employing a mixed SAM strategy that combined cysteine-tagged Protein G and 11-mercaptoundecyl-tri(ethylene glycol) (OEG), achieving a detection limit of 100 fM.

However, EGOFETs based on mixed SAM modification still face several challenges in practical applications [29,30,31,32]. An ideal SAM should provide a high density of reactive groups (e.g., -COOH [33,34]) and promote the oriented, ordered arrangement of antibodies with their antigen-binding (Fab) regions facing outward [35,36,37,38,39]. Poorly designed or disordered SAMs can lead to random “lying-down” [40] or aggregation [41] of antibodies, which masks their binding sites and directly reduces their capacity to capture antigens effectively [35,42,43,44]. Furthermore, studies indicate that defects within the SAM, such as disordered patches resulting from oxidation [45], can increase nonspecific adsorption of antibodies. These defects also reduce the density and accessibility of available carboxyl groups for antibody immobilization, consequently leading to compromised sensitivity [46,47,48].

Herein, we report the tuning of growth and distribution of SAM on the gold gate surface through concentration modulation (10–400 mM) to enhance the effective immobilization of antibody-based biorecognition elements. The optimization leads to achievement of high-performance sensing of IgG based on EGOFET. Leveraging the excellent signal amplification capability of EGOFETs, highly sensitive, specific, and linear detection in a wide range of IgG has been achieved. This study reveals a non-monotonic dependence of sensor performance on SAM concentration: either too low or too high a thiol concentration hinders uniform SAM diffusion, adsorption, and growth on the surface, thereby impairing antibody grafting and degrading device sensing performance. The SAM quality-control strategy established here can be extended to highly sensitive detection systems for other disease biomarkers, offering a universal interfacial-engineering approach for developing modular and scalable EGOFET-based biosensors.

## 2. Materials and Methods

### 2.1. Materials

11-mercaptoundecanoic acid (11-MUA), 3-mercaptopropionic acid (3-MPA), phosphate-buffered saline (PBS), 1-ethyl-3-(3-dimethylaminopropyl)carbodiimide hydrochloride (EDC), anti-IgG, bovine serum albumin (BSA), Immunoglobulin A (IgA), and Immunoglobulin M (IgM) were purchased from Sigma-Aldrich, Shanghai, China; Chloroform, acetone, and anhydrous ethanol were purchased from Sinopharm Chemical Reagent Co., Ltd., Shanghai, China. Regioregular poly(3-hexylthiophene) (P3HT, >95% regioregularity, average molecular weight of 36–58 kDa) was purchased from RIKEN, Wako, Saitama, Japan. N-hydroxysuccinimide (NHS) was purchased from Shanghai Yuanye Bio-Technology Co., Ltd., Shanghai, China; Ethanolamine was purchased from Aladdin Reagent Co., Ltd., Shanghai, China; IgG was purchased from Yisheng Biotechnology (Shanghai) Co., Ltd., Shanghai, China. Ultrapure water (18.2 MΩ·cm) was obtained from a Milli-Q water purification system (Merck KGaA, Darmstadt, Germany).

### 2.2. EGOFET Fabrication

Source and drain electrodes were patterned on a silicon/silicon dioxide (Si/SiO_2_) substrate using standard photolithography. A bilayer of Cr (3 nm, deposited at 0.1 Å/s) and Au (40 nm, deposited at 0.3 Å/s) was deposited to form the contacts, defining a channel with a width-to-length ratio (W/L) of 1340 μm/10 μm. Subsequently, the semiconductor channel was formed by spin-coating a solution of P3HT/Chloroform (6 mg/mL) at 2000 rpm for 30 s onto the base electrode, followed by thermal annealing at 125 °C for 30 min. The gate electrode was fabricated by thermal evaporation of Au (50 nm thick) with an effective area of approximately 0.6 cm^2^. Finally, PBS electrolyte was introduced onto the channel to establish contact with the gate, enabling electrostatic modulation of the semiconducting channel by the gate electrode.

### 2.3. Gate Bio-Functionalization

The gold gate surface was functionalized for specific analyte recognition through the following steps. First, the gate was cleaned by sequential sonication in acetone, ethanol, and isopropanol (10 min each), followed by oxygen plasma treatment (100 W, 3 min). A mixed self-assembled monolayer was then formed by immersing the cleaned gate in an ethanolic solution of 3-MPA and 11-MUA (molar ratio = 10:1) at concentrations ranging from 10 mM to 400 mM. The concentration refers to the total concentration of the mixed thiols (3-MPA and 11-MUA) in the ethanol solution. For example, in the 50 mM mixed SAM solution, the actual concentration of 3-MPA was approximately 45.45 mM and that of 11-MUA was approximately 4.55 mM. This was prepared by dissolving 2.412 g of 3-MPA and 0.4965 g of 11-MUA in 500 mL of ethanol.

The assembly was carried out overnight in the dark under N_2_ at room temperature. After rinsing with ethanol and ultrapure water, the carboxyl termini were activated by incubating the gate in an aqueous solution of EDC (200 mM) and NHS (50 mM) for 2 h at room temperature. The biorecognition layer was subsequently grafted by incubating the activated gate in an anti-IgG solution (0.1 mg/mL in 1× PBS) for 2 h, enabling covalent coupling between the antibody amine groups and the activated carboxyl sites. To block unreacted sites, the gate was then transferred to 1 M ethanolamine in 1× PBS for 1 h, followed by an additional 1 h in 1× PBS containing 0.1 mg/mL BSA at 25 °C to minimize nonspecific adsorption. After each functionalization step, the gate was rinsed thoroughly with ultrapure water.

### 2.4. Sensing Measurements

The electrical characterization of the EGOFETs was performed at room temperature in ambient air using a semiconductor parameter analyzer (JJS-BE-150, Primarius Technologies Co., Ltd., Shanghai, China). PBS solution served as the electrolyte, with its contact area confined by a patterned polydimethylsiloxane (PDMS) well. Output characteristics parameters: V_G_ = 0 to −0.6 V, with a step size of −0.1 V. Transfer characteristics parameters: V_D_ = −0.6 V, V_G_ = 0.2 to −0.6 V, with a step size of −0.02 V. All sensing experiments were conducted after the device electrical performance had stabilized. For the sensing measurements, PBS solutions containing different concentrations (10^−15^ M to 10^−9^ M) of IgG were introduced into the electrolyte chamber. After a 10 min incubation in the dark at room temperature, the transfer characteristics were recorded.

### 2.5. Gate Characterization

The gate surface morphology was characterized using Atomic Force Microscopy (AFM) (Bruker Dimension Icon) (Bruker Corporation, Billerica, MA, USA) and Scanning Electron Microscopy (SEM) (ZEISS G500) (Carl Zeiss AG, Oberkochen, Germany). X-ray Photoelectron Spectroscopy (XPS) was performed using an XPS spectrometer (Shimadzu Corporation, Kyoto, Japan). Attenuated Total Reflection (ATR) infrared spectroscopy was conducted with a Fourier transform infrared spectrometer (FTIR) (Bruker Corporation, Billerica, MA, USA). Cyclic Voltammetry (CV) and Electrochemical Impedance Spectroscopy (EIS) were measured using the Biologic-Ec-lab SP 150 electrochemical workstation (Seyssinet-Pariset, France).

### 2.6. Computing the LOD

The LOD was defined as the concentration of IgG at which the elicited response was (ΔI/I_0_)_mean_ + 3σ [27,49], where (ΔI/I_0_)_mean_ is the average current response of the blank control, and σ is the standard deviation.

### 2.7. Version Number of Software

The following software versions were used: OriginPro 2021 (9.8.0.200), EC-Lab V11.52, Avantage 5.9931, ChemDraw 20.0.0.41, and NanoScope Analysis 2.0.

## 3. Results

**Figure 1:**
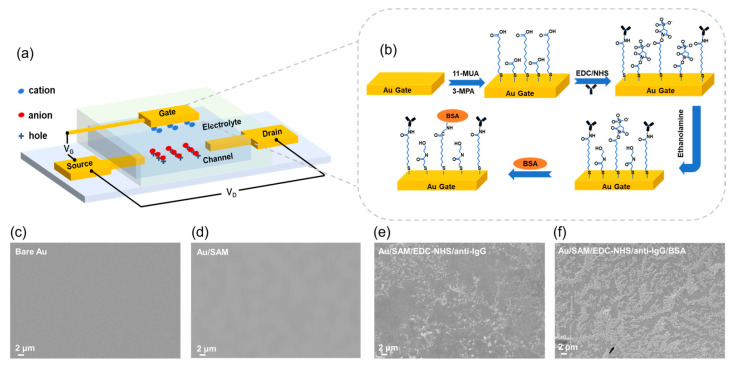
Schematic illustration of the EGOFET-based IgG sensor. (**a**) Device configuration. (**b**) Functionalization process on the gate electrode. (**c**–**f**) SEM images showing the gate surface after each modification step: (**c**) bare Au, (**d**) Au/SAM, (**e**) Au/SAM/EDC-NHS/anti-IgG, and (**f**) Au/SAM/EDC-NHS/anti-IgG/BSA. Scale bars, 2 μm.

The device configuration of the EGOFET biosensor is illustrated in Figure 1a. The device is constructed on a Si/SiO_2_ substrate and comprises Au/Cr source/drain electrodes, a P3HT semiconductor channel, a PBS electrolyte, and a functionalized gold gate. As schematically depicted in Figure 1b, the gate surface is biofunctionalized to enable specific target recognition: a SAM provides covalent anchoring sites for the anti-IgG antibodies. Upon binding of IgG from the sample solution, the signal generated by this recognition event is transduced to the functional surface, which in turn modulates the channel current.

Surface morphology before and after the modification steps reveals an extensive distribution of immobilized substances (Figure 1c–f). Figure 1c shows the surface morphology of the bare gold gate electrode, which appears relatively smooth and flat, free from obvious impurities or defects. Figure 1d presents the surface morphology after the formation of the SAM. As the SAM is an ultrathin and flat film with a molecular-scale thickness, no significant morphological change is observed compared to the bare gold electrode. Figure 1e displays the surface morphology after antibody immobilization. A large number of scattered attachments are observed on the surface, exhibiting typical characteristics of biomolecular loading, which confirms the successful immobilization of antibodies onto the electrode surface. Figure 1f shows the surface morphology following blocking with BSA. The surface attachments are further increased, demonstrating successful surface modification with biomolecules.

To assess the quality of the SAM and its subsequent influence on antibody immobilization, which directly modulates the sensing performance, the concentration of the assembly solution was varied from 10 mM to 400 mM.

**Figure 2:**
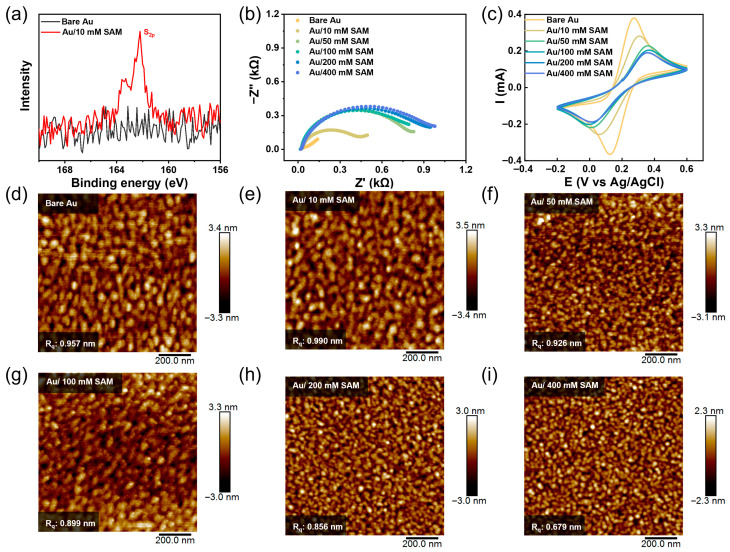
Characterization of Au gates modified with SAM at different concentrations (0\10\50\100\200\400 mM). (**a**) XPS high-resolution spectra of the S 2p core level, (**b**) Nyquist plot, (**c**) cyclic voltammograms, and (**d**–**i**) AFM images.

The functionalized gate surfaces were first analyzed by XPS to systematically evaluate these effects. The high-resolution XPS spectrum of the S 2p core level is shown in Figure 2a. Compared to the bare gold gate (black curve), a distinct S 2p signal emerged at a binding energy of approximately 162.0 eV after modification with a 10 mM thiol solution (red curve). This signal can be fitted into two peaks: S 2p_3/2_ and S 2p_1/2_, separated by 1.17 eV with an area ratio of about 2:1, consistent with the expected spin–orbit splitting for sulfur. The absence of significant spectral features in the higher binding energy region (166–170 eV) indicates that the thiolates were not oxidized. These results confirm the successful grafting of thiol molecules onto the gold surface via Au-S bonds [50,51,52,53,54]. Analysis of the SAM formed at higher thiol concentrations (50 and 100 mM) reveals a decrease in S 2p signal intensity with higher thiol concentration. As shown in Figure S1, at 50 mM, the characteristic doublet (S 2p_3/2_ and S 2p_3/2_) remained discernible, although attenuated. In contrast, at 100 mM, the distinct double-peak structure was largely lost, leaving a broadened S 2p envelope. This trend suggests that at a low concentration (10 mM), a sparse distribution of thiol molecules allows clear detection of well-defined Au-S bonds. The persistence of the bimodal signature at 50 mM indicates that the molecules maintain a relatively ordered arrangement, while the signal attenuation likely reflects increased packing density. At 100 mM, however, excessive molecular adsorption appears to create a disordered and inhomogeneous interfacial environment, resulting in the loss of spectral resolution while the overall sulfur signal remains detectable.

To further investigate the properties of the self-assembled layer functionalized gold gate surfaces, electrochemical characterization was performed. Figure 2b,c shows EIS and CV curves in a 5 mM [Fe(CN)_6_]^3−/4−^ PBS electrolyte system. The Nyquist plot displayed a marked increase in the semicircle radius with higher thiol concentration, reflecting a growing barrier to charge transfer at the electrolyte–gate interface [55,56,57]. Correspondingly, the interfacial charge-transfer resistance (R_ct_) of the bare gold gate (2.73 × 10^−3^ Ω) rose to 473 Ω after modification at 10 mM and further increased to 828 Ω at 50 mM (Figure S2a). The larger R_ct_ at 50 mM suggests a denser and more insulating SAM formed, which hinders the diffusion of redox ions toward the electrode surface. A similar trend was observed in the CV curves. The electrochemical oxidation peak shifted from 272 mV (bare gold) to 304 mV (10 mM SAM) and 356 mV (50 mM SAM), while the peak-to-peak separation (ΔV_P_) increased from 146 mV to 246 mV and 349 mV, respectively (Figure S2b). Above 50 mM (up to 400 mM), further increases in thiol concentration produced only minor changes in peak position, indicating that a densely packed SAM had essentially formed at 50 mM. Higher concentrations likely only filled residual defects in the existing monolayer, with limited additional effect on interfacial electrochemistry.

AFM images (Figure 2d–i) revealed that the introduction of the SAM increased surface roughness relative to bare gold, but roughness subsequently decreased at higher modification concentrations. This suggests that at elevated concentrations, excess thiol molecules physically adsorb into remaining vacancies, thereby enhancing the macroscopic surface flatness of the monolayer. Based on the above results, it is suggested that at a thiol concentration of 10 mM, the SAM grows freely but exhibits low surface coverage and packing density. At 50 mM, a stable and densely packed SAM forms at the gate interface. However, when the concentration exceeds 100 mM, excessively high thiol levels lead to uneven molecular diffusion and distribution on the surface, thereby compromising the structural integrity of the monolayer.

**Figure 3:**
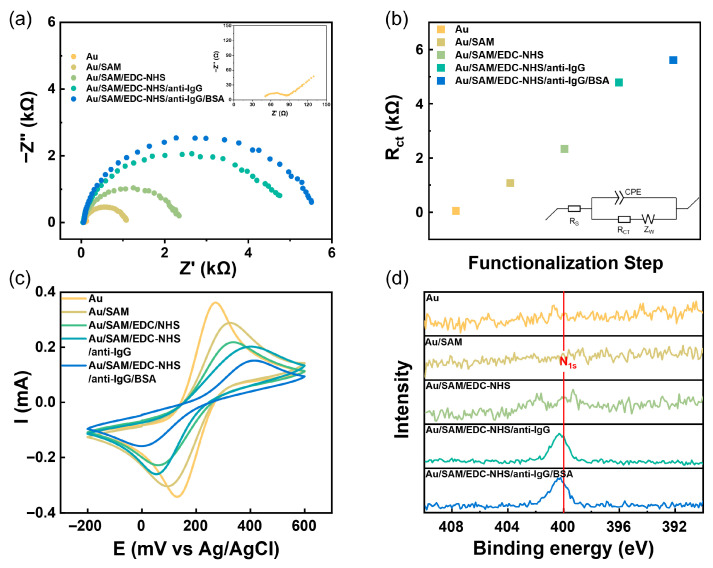
Characterization of the Au gate after sequential functionalization steps. (**a**) Nyquist plot, (**b**) equivalent circuit models and the calculated value of the R_ct_, (**c**) CV, and (**d**) XPS high-resolution spectra of the N 1s core level.

To quantitatively analyze the impact of different functionalization stages on electron transfer at the surface interface, EIS measurements were conducted. Taking 50 mM SAM-modified gate as an example, the impedance evolution was recorded in a 5 mM [Fe(CN)_6]_^3−/4−^ PBS electrolyte after each modification step: bare gold gate (Au), after SAM formation (Au/SAM), following carboxyl activation with EDC/NHS (Au/SAM/EDC-NHS), after anti-IgG immobilization (Au/SAM/EDC-NHS/anti-IgG), and after complete functionalization (Au/SAM/EDC-NHS/anti-IgG/BSA). The Nyquist plots (Figure 3a) showed a progressive increase in the semicircle diameter with each added layer, indicating rising charge-transfer resistance. For quantitative analysis, the impedance data were fitted using an equivalent circuit comprising solution resistance (R_s_), charge-transfer resistance (R_ct_), a constant-phase element (CPE), and Warburg impedance (Z_W_). The fitted R_ct_ values (Figure 3b) confirm that each modification step successfully altered the interface, with the anti-IgG immobilization step contributing the largest increase (ΔR_ct_ ≈ 2453 Ω). The corresponding CV curves (Figure 3c) further supported these findings, showing a sequential decrease in peak current and a positive shift in oxidation peak potential, further confirming the effective modification achieved by the multi-layer functionalization process [10,27,58].

High-resolution N 1s XPS spectra were collected at each functionalization step to verify the chemical composition of the modified interface (Figure 3d). After EDC/NHS activation, the emergence of an N 1s signal was clearly detected. This signal intensified significantly following anti-IgG immobilization, confirming the successful grafting of antibodies onto the surface. Subsequent blocking by ethanolamine and BSA resulted in only minor spectral changes, consistent with the expected completion of the functionalization process. Changes in surface roughness, as revealed by AFM, further corroborated the stepwise modification (Figure S3). The introduction of the SAM and the antibody layer led to a marked increase in surface roughness. In contrast, the block with ethanolamine and BSA filled defects caused by SAM modification and antibody immobilization, yielding a smoother surface [59]. Additionally, ATR spectroscopy of the antibody-modified gate displayed characteristic amide absorptions at 1680–1630 cm^−1^ (amide I), 1640–1550 cm^−1^ (amide II), and a broad N-H/O-H stretching band at 3500–3100 cm^−1^ (Figure S4), providing independent evidence of successful antibody immobilization. In summary, the sequential biofunctionalization was successfully achieved on the SAM-modified gate. To probe the influence of SAM quality on device performance, functionalized gates prepared with 10 mM, 50 mM, and 100 mM thiol solutions were subsequently integrated into EGOFET devices for electrical characterization.

**Figure 4:**
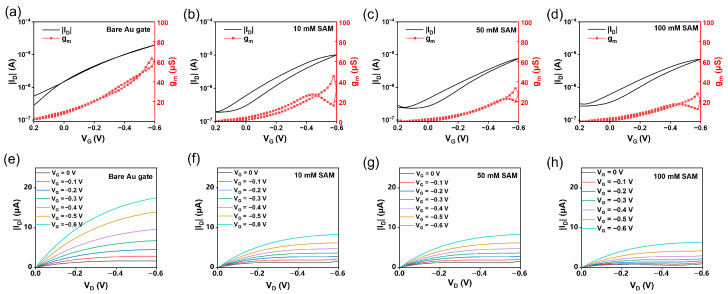
The basic electrical properties of the bare Au gate and the functionalized Au gate (10\50\100 SAM-modified). (**a**–**d**) Transfer characteristics of the EGOFETs under different V_G_ conditions, (**a**) bare Au and functionalized Au gate prepared with (**b**) 10 mM, (**c**) 50 mM, and (**d**) 100 mM SAM. (**e**–**h**) Output characteristics of the EGOFETs at varying gate voltages (V_G_, 0 to −0.6 V) and drain voltages (V_D_, 0 to −0.6 V), (**e**) bare Au and functionalized Au gate prepared with (**f**) 10 mM, (**g**) 50 mM, and (**h**) 100 mM SAM.

Figure 4 presents the transfer and output characteristics of EGOFETs employing P3HT as the channel material measured in 1× PBS electrolyte with different gate electrodes.

As shown in Figure 4a–d, the device with a bare gold gate exhibited excellent electrical performance, characterized by a high channel current, large peak current, narrow hysteresis window, and high transconductance, indicating efficient gate modulation. In contrast, devices with functionalized gates showed a significantly enlarged hysteresis window. With increasing SAM modification concentration, both the peak current and transconductance displayed a systematic decreasing trend (Table S1). Furthermore, the output characteristics (Figure 4e–h) revealed that the drain currents were substantially reduced at all gate voltages for functionalized gates, further confirming the weakened gate modulation. These performance changes are primarily attributed to the introduction of a higher charge-transfer resistance at the gate interface by the functionalization layers, which reduces the effective gate voltage actually applied to the semiconductor channel.

**Figure 5:**
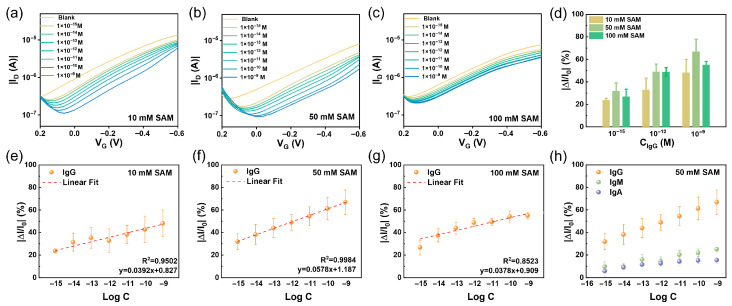
Performance of the EGOFET-based IgG sensors. (**a**–**c**) The I_D_ changes of the IgG gate (**a**) 10 mM, (**b**) 50 mM, (**c**) 100 mM SAM in response to varying concentrations of target molecules. (**d**) Comparison of the NR value of the functionalized Au gate at different concentrations of the IgG (10^−15^ M, 10^−12^ M, 10^−9^ M). Data are presented as the mean ± SD (n = 3). (**e**–**g**) Normalized sensor response of functionalized Au gates (**e**) 10 mM, (**f**) 50 mM, (**g**) 100 mM SAM to the specific target (IgG), the NR value is calculated at V_G_ = −0.6 V. Data are presented as the mean ± SD (n = 3). (**h**) The NR value for specific targets (IgG) and non-specific targets (IgA and IgM). Data are presented as the mean ± SD (n = 3).

To assess the impact of SAM interface quality on sensing performance, EGOFETs fabricated with functionalized gates (10, 50, and 100 mM SAM-modified) were used to detect varying concentrations of IgG. The results are summarized in Figure 5. Despite the different thiol concentrations used for SAM formation, all devices exhibited a consistent response: as the target concentration increased, the transfer curves shifted toward more negative gate voltages, and the peak current decreased progressively (Figure 5a–c). This behavior aligns with previously reported trends [7,27,60,61] and can be attributed to the specific binding of IgG to the immobilized anti-IgG on the gate surface. The formation of an insulating protein layer increases the interfacial resistance, a mechanism directly supported by EIS. As shown in Figure S5, the diameter of the Nyquist plot expanded with increasing IgG concentration, corresponding to an increase in interfacial charge-transfer resistance.

For the quantitative evaluation of the sensing performance of devices based on SAM with different concentrations (10, 50, and 100 mM), the normalized current change at a gate voltage of V_G_ = −0.6 V was extracted for analysis. The normalized response (NR) is defined as

NR = (I_0_ − I)/I_0_, (1)

where I_0_ is the baseline current. As shown in Figure 5e–g, devices based on the 50 mM SAM-modified gate exhibit the ultimate linear response (R^2^ = 0.998), with a detection sensitivity of 5.78% per decade for IgG and LOD as low as 2.82 fM, demonstrating that the device possesses high sensitivity, a linear response profile, and robust quantitative detection capability. Compared with previously reported studies (Table 1), this work achieves a broad detection range of 1 fM–10 nM and attains a lower LOD simultaneously.

To contextualize the analytical performance achieved here, we also compared the LOD with the clinically relevant concentration range of IgG. It is worth noting that the normal concentration of IgG in human serum typically ranges from approximately 7 to 16 mg/mL (about 46.7–106.7 nM). The LOD achieved in this work is 2.82 fM, which is more than five orders of magnitude lower than the clinically relevant concentration range.

To further compare the influence of SAM quality on sensing performance, the NR values for low (10^−15^ M), medium (10^−12^ M), and high (10^−9^ M) concentrations of IgG were extracted and compared (Figure 5d). Under all three modification conditions, the NR values increased monotonically with IgG concentration. Notably, the 50 mM SAM groups yielded the highest NR values at each concentration point. This non-monotonic dependence of the sensing performance on SAM concentration reflects the critical impact of interfacial quality on subsequent biofunctionalization. The incomplete monolayer formed at 10 mM, characterized by low coverage and density as well as abundant defects, resulted in limited antibody loading and thus a weaker target response. In contrast, the stable, well-ordered, and densely packed SAM achieved at 50 mM provided an optimal platform for high-density and oriented antibody immobilization, leading to the highest detection sensitivity and sensing response. At 100 mM, excessive thiol concentration likely induced physical adsorption and disordered molecular packing, which impaired the subsequent functionalization (activation and antibody grafting) of the terminal carboxyl groups. This reduced the number of available antigen-binding sites and consequently led to slightly inferior sensing performance.

Furthermore, the response of the functionalized gate (50 mM SAM) to non-specific targets, IgA and IgM, was evaluated to check the specificity property of the functionalization. As shown in Figure 5h, the normalized response for the specific target IgG was significantly higher than that for IgA and IgM. The signals generated by the latter two were substantially lower than the specific signal (the corresponding current responses are provided in Figure S6). These results confirm the excellent specificity of the fabricated functionalized gate toward IgG. As a control, the response of the bare gold gate to IgG was also evaluated (Figure S7), showing no significant current change upon target addition, confirming that the specific signal originates from the biofunctionalized interface.

## 4. Conclusions

In summary, this work achieves effective regulation of SAM growth and quality on the gold gate electrode surface through thiol concentration modulation (from 10 mM to 400 mM), thereby influencing the subsequent antibody grafting efficiency. A well-ordered, stable, and densely packed SAM was achieved at an optimized mixed thiol concentration of 50 mM. This optimal interface not only provides abundant accessible binding sites for antibodies but also facilitates orderly and stable charge transfer across the electrode/electrolyte interface. Although the fully functionalized gate exhibited reduced modulation efficiency compared to bare gold, the resulting biosensor demonstrated a highly linear response (R^2^ = 0.998) over a wide detection range spanning 6 orders of magnitude and a limit of detection as low as 2.82 fM, together with excellent selectivity. It should be noted that the sensing performance of our thiol-based mixed SAM system is lower than that reported in [27], of which the hydrogen-bonding network within the amide-containing SAM enables long-range electrostatic cooperative interactions under an applied gate electric field. A single binding event propagates local defects across the entire SAM, causing a substantial decrease in the gate work function—amplifying a nanoscale binding event into a macroscopic signal detectable at the millimeter scale. This signal amplification mechanism accounts for the ultrahigh sensitivity down to the single-molecule level [27]. Comparing our thiol-based mixed SAM system with that in [27], the two strategies offer distinct advantages suited to different applications. The hydrogen-bonding approach achieves exceptional sensitivity through signal amplification, making it ideal for single-molecule detection in low-dose scenarios. In contrast, our strategy focuses on optimizing SAM quality via systematic thiol concentration tuning, delivering a significantly wider linear detection range while maintaining high sensitivity—offering more balanced performance with broad dynamic range and robust quantification across multiple concentration decades.

Furthermore, it is worth noting that although the EDC/NHS coupling method used in this study results in randomly oriented antibodies, the optimized SAM concentration still enabled satisfactory sensor performance. We note that oriented immobilization using protein A or protein G could potentially enhance antigen binding efficiency, and this strategy should be explored in future work.

In addition, although this work is promising for highly sensitive sensing, further studies are required to meet the demands of practical clinical applications, such as validation in complex biological matrices (e.g., serum or whole blood), device-to-device uniformity and long-term reliability, and integration into portable point-of-care platforms. Addressing these challenges will enable EGOFET-based biosensors to play a key role in the early diagnosis of IgG-related diseases and other biomarkers.

## Figures and Tables

**Table 1:** Summary of typical protein sensors based on EGOFETs and electrochemical sensors.

Category	Modification	Analyte	LOD	Detection Range	Ref.
Electrochemical sensors	GCE/PEDOT-citrate/AuNPs/Y-shaped peptide ^(a)^	IgG	213 fM	667 fM–66.7 pM	[62]
Electrochemical sensors	MIPs NPs ^(b)^/MoS2@N-GQDs-IL/GCE	IgG	133 fM	667 fM–333 pM	[1]
Electrochemical sensors	MIP/CS ^(c)^/Cu-MOF/GCE	IgG	20 fM	66.7 fM–66.7 pM	[63]
EGOFETs	Au/SAM/antibody	α-synuclein	0.25 pM	25 pM–25 nM	[64]
EGOFETs	Au/SAM/anti-IgG	IgG	Single protein	6 × 10^−2^ zM–6 × 10^8^ zM	[27]
EGOFETs	Au/PEG/aptamer	Ricin	30 pM	30 pM–300 nM	[65]
EGOFETs	Au/protein G/OEG ^(d)^/Nivolumab	ADA	100 fM	1 pM–10 nM	[28]
EGOFETs	Au/Protein-G/anti-NF-L	NF-L ^(e)^	30 fM	100 fM–10 nM	[66]
EGOFETs	Au/OEG/anti-THFα	THFα ^(f)^	3 pM	1 pM–10 nM	[67]
EGOFETs	Au/Protein G/anti-IL6	IL6	1 pM	1 pM–10 nM	[68]
EGOFETs	P3HT/anti-PCT	PCT ^(g)^	2.2 pM	0.8 pM–4.7 nM	[12]
EGOFETs	P3HT/anti-IgG	IgG	2.9 pM	4 pM–4 × 10^6^ pM	[49]
EGOFETs	P3HT/anti-CPR	CPR	2 pM	4 pM–2 μM	[69]
EGOFETs	Au/SAM/anti-IgG	IgG	2.82 fM	1 fM–10 nM	This work

^(a)^ Glassy carbon electrode/PEDOT-citrate/gold nanoparticles/Y-shaped peptide (CPPPPEK (HWRGWVA) EKEKE); ^(b)^ CuFe2O4 molecularly imprinted polymer nanoparticles; ^(c)^ chitosan; ^(d)^ 11-mercaptoundecyltriethylene glycol; ^(e)^ neurofilament light chain; ^(f)^ proinflammatory cytokine tumor necrosis alpha; ^(g)^ procalcitonin.

## Data Availability

Data are contained within the article and Supplementary Materials.

## Supplementary Materials

The following supporting information can be downloaded at: https://www.mdpi.com/article/10.3390/s26082290/s1. Figure S1: XPS high-resolution spectra of the S 2p core level; Figure S2: Characterization of the Au gate modified with SAM at different concentrations (0\10\50\100\200\400 mM). (a) Equivalent circuit models and the calculated value of the R_ct_, and (b) peak separation ΔV_p_ in CV; Figure S3: AFM images of the Au gate after sequential functionalization steps. (a) bare Au gate, R_q_ = 0.957 nm, (b) Au/SAM gate, R_q_ = 0.990 nm, (c) Au/SAM/EDC-NHS/anti-IgG gate, R_q_ = 1.15 nm, and (d) Au/SAM/EDC-NHS/anti-IgG/MEA/BSA gate, R_q_ = 0.860 nm; Figure S4: FTIR-ATR spectra of functionalized Au gate (Au/SAM/EDC-NHS/anti-IgG); Figure S5: Characterization of Au gate modified with 50 mM SAM to varying concentrations of target molecules. (a) Nyquist plot and (b) Equivalent circuit models and the calculated value of the R_ct_; Figure S6: The selectivity of the EGOFET-based IgG sensors. (a,b) The I_D_ changes of the IgG gate (50 mM SAM) in response to varying concentrations of (a) IgM and (b) IgA; Figure S7: Response of the Au gate to varying concentrations of IgG. (a) The I_D_ changes and (b) the NR value of the Au gate in response to varying concentrations of IgG; Table S1: Summary of the basic electrical performance parameters of functionalized Au gate modified with SAM at different concentrations (0\10\50\100 mM). Data are presented as mean ± SD (n = 3).
