# Supplementary material for: Interface Quality Control of Self-Assembled Monolayer for Highly Sensitive Protein Detection Based on EGOFETs
Source: Sensors (Basel). 2026 Apr 8;26(8):2290. doi: 10.3390/s26082290 (PMC13120370; doi:10.3390/s26082290)
Supplement: Supplementary file 1 [file sensors-26-02290-s001.zip › sensors-4225265-supplementary.pdf]

Interface quality control of self-assembled monolayer for high sensitive protein detection based on EGOFETs

Xinyu Dong [1], Xingyu Jiang [1], Jiaqi Su [2], Zhongyou Lu [1], Cheng Shi [3], Dianjue Liu [1], Lizhen Huang [1,*] and Lifeng Chi [1,3,*]

[1] State Key Laboratory of Bioinspired Interfacial Materials Science, Institute of Functional Nano & Soft Materials (FUNSOM), Soochow University, Suzhou 215123, China; 20234214162@stu.suda.edu.cn(X.D.); xyjiang@suda.edu.cn(X.J.); zyluzy@stu.suda.edu.cn(Z.L.); shicheng1708404032@163.com(C.S.); djliu2819@stu.suda.edu.cn(D.L)

[2] School of Integrated Circuits, Southeast University, Nanjing, China; 220236459@seu.edu.cn(J.S.)

[3] Macao Institute of Materials Science and Engineering (MIMSE), MUST-SUDA Joint Research Center for Advanced Functional Materials, Macau University of Science and Technology, Taipa 999078, Macao, China

* Correspondence: lzhuang@suda.edu.cn(L.H.); chilf@suda.edu.cn(L.C.)

Supplementary Figures

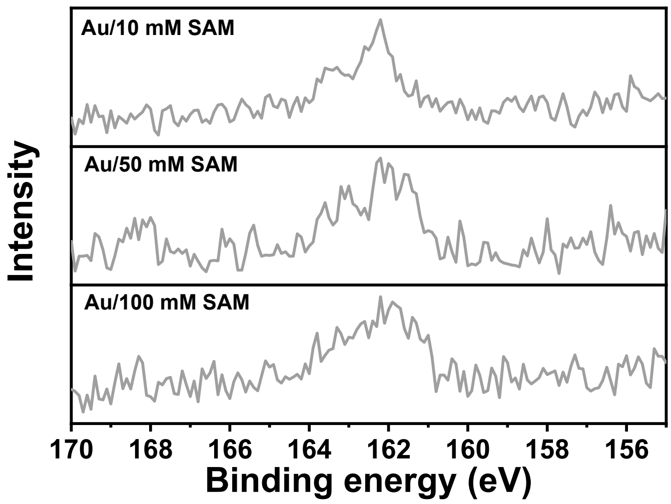

Figure S1. XPS high-resolution spectra of the S 2p core level.

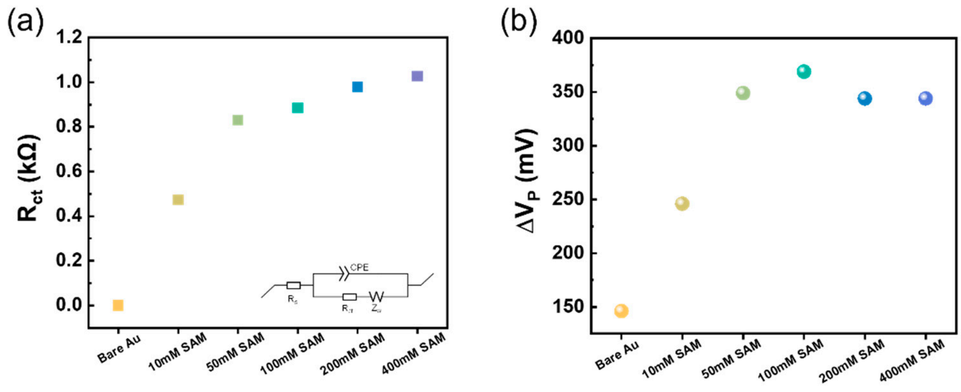

Figure S2. Characterization of the Au gate modified with SAM at different concentrations (0\10\50\100\200\400 mM). (**a**) Equivalent circuit models and the calculated value of the R_{ct}, and (**b**) peak separation ΔV_p in CV.

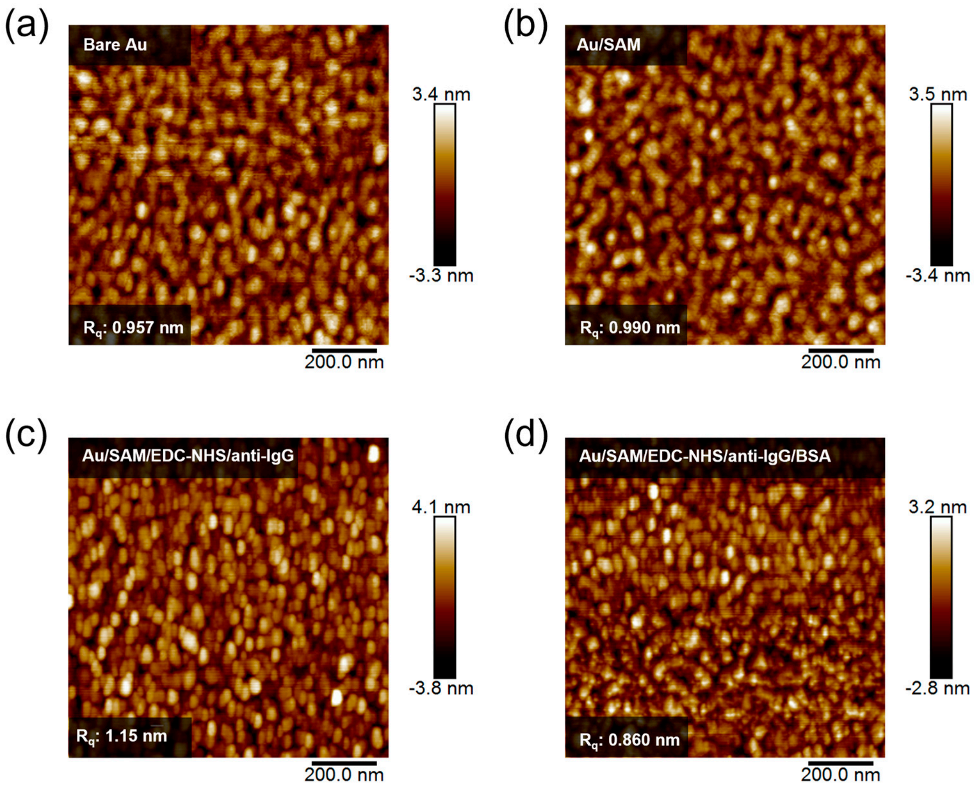

Figure S3. AFM images of the Au gate after sequential functionalization steps. (**a**) bare Au gate, R_q = 0.957 nm, (**b**) Au/SAM gate, R_q = 0.990 nm, (**c**) Au/SAM/EDC-NHS/anti-IgG gate, R_q = 1.15 nm, and (**d**) Au/SAM/EDC-NHS/anti-IgG/MEA/BSA gate, R_q = 0.860 nm.

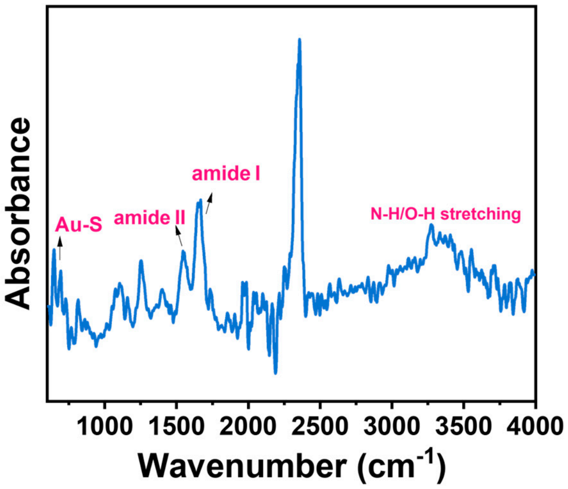

Figure S4. FTIR-ATR spectra of functionalized Au gate (Au/SAM/EDC-NHS/anti-IgG).

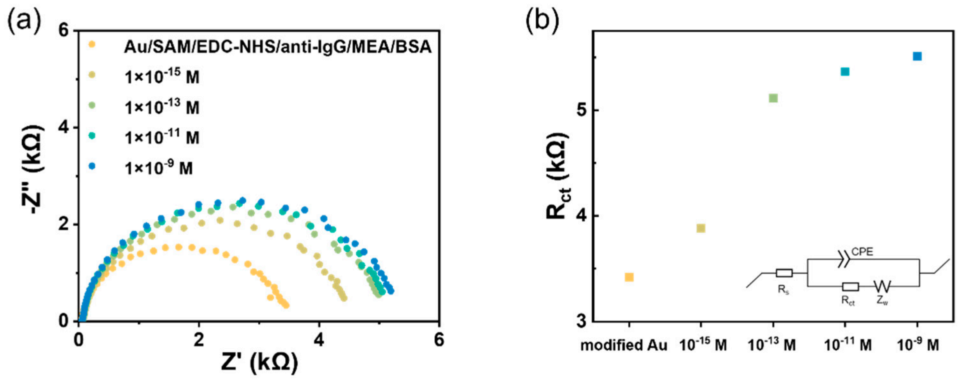

Figure S5. Characterization of Au gate modified with 50 mM SAM to varying concentrations of target molecules. (**a**) Nyquist plot and (**b**) Equivalent circuit models and the calculated value of the R_{ct}.

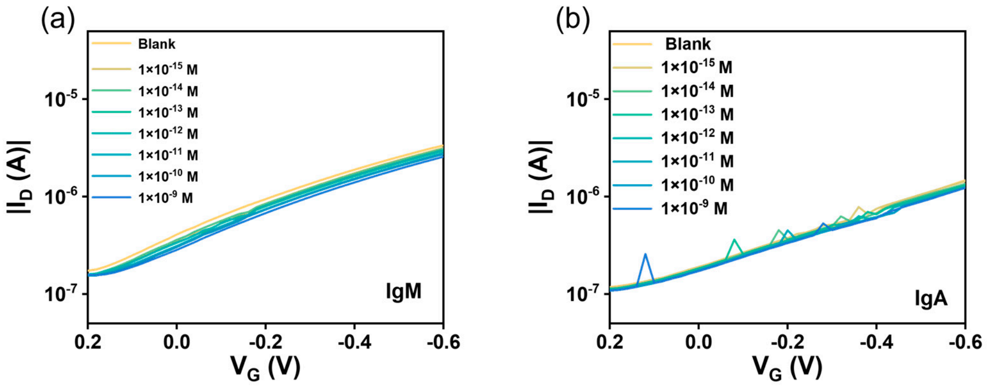

Figure S6. The selectivity of the EGOFET-based IgG sensors. (**a-b**) The I_D changes of the IgG gate (50 mM SAM) in response to varying concentrations of (**a**) IgM and (**b**) IgA.

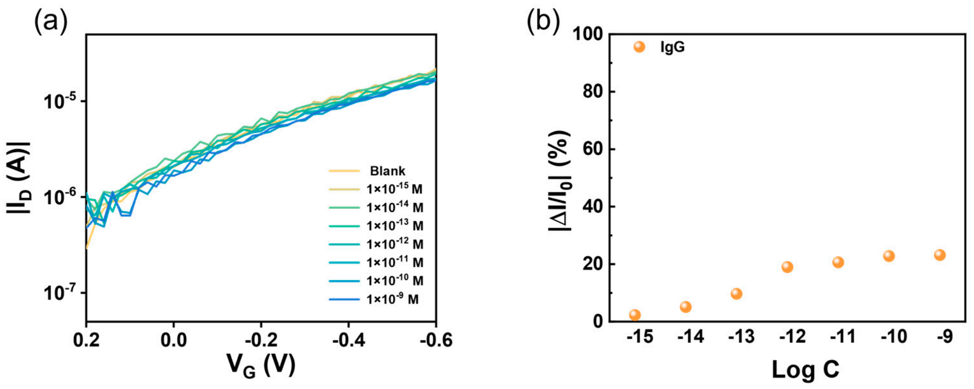

Figure S7. Response of the Au gate to varying concentrations of IgG. (**a**)The I_D changes and **(b)**the NR value of the Au gate in response to varying concentrations of IgG.

Supplementary Tables

Table S1. Summary of the basic electrical performance parameters of functionalized Au gate modified with SAM at different concentrations (0\10\50\100 mM). Data are presented as mean ± SD (n = 3).

	g_m (μS)	I_D (μA) (V_G = -0.6 V)
Au	69.5 ± 15.7	24.1 ± 4.8
10 mM 11-MUA/3-MPA	39.5 ± 8.6	14.7 ± 5.2

50 mM 11-MUA/3-MPA	29.2 ± 5.2	11.1 ± 3.6
100 mM 11-MUA/3-MPA	18.5 ± 4.2	6.6 ± 2.9
